# Effects of Regioselectivity and Lipid Class Specificity of Lipases on Transesterification, Exemplified by Biodiesel Production

**DOI:** 10.1007/s11746-014-2465-7

**Published:** 2014-04-26

**Authors:** Dovilė Šinkūnienė, Patrick Adlercreutz

**Affiliations:** 1 Department of Biotechnology, Lund University, P.O. Box 124, SE-221 00 Lund, Sweden; 2 Present Address: Department of Biochemistry and Molecular Biology, Vilnius University, M. K. Čiurlionio g. 21, 03101 Vilnius, Lithuania

**Keywords:** Biodiesel, Lipase, Transesterification, Alcoholysis, Acyl migration

## Abstract

Lipase-catalyzed ethanolysis of triolein was studied as a model for biodiesel production. Four lipases were immobilized on porous polypropylene, and ethanolysis reactions were carried out in methyl *t*-butyl ether. The reaction products were analyzed using gas chromatography. Three of the four lipases studied were efficient in the conversion of triolein to 2-monoolein, but slow in the final step of producing glycerol. However, *Candida antarctica* lipase B was slow in the conversion of triolein, but more efficient in the subsequent two steps than the other lipases. The 1,3-selectivity of the lipases was less pronounced for the monooleins than for triolein. Silica gel was investigated as a catalyst for acyl migration, showing an increase in biodiesel yield with three of the lipases, but a reduction in yield when *C. antarctica* lipase B was used. The highest biodiesel yield (96 %) was obtained with a combination of *Rhizopus arrhizus* lipase and *C. antarctica* lipase B.

**Electronic supplementary material:**

The online version of this article (doi:10.1007/s11746-014-2465-7) contains supplementary material, which is available to authorized users.

## Introduction

Lipase-catalyzed transesterification between triacylglycerols and short-chain alcohols is an attractive way of producing fatty acid esters for various applications. Esters of health-promoting fatty acids are used as nutraceuticals, with long-chain omega-3 fatty acid esters as a typical example. The main constituent of biodiesel is alkyl esters, most commonly methyl esters. Interest in biodiesel has increased rapidly due to the increase in demand for renewable fuels. Many types of fats and oils can be used as raw materials [1], and large-scale production is currently carried out mainly as a chemically catalyzed alcoholysis reaction [2, 3]. Enzymatic alcoholysis is an interesting alternative and has been shown to be especially beneficial when raw materials rich in free fatty acids are used, since they can be used directly in the enzymatic process, while pretreatment of such substrates is needed prior to the corresponding chemical conversion [2]. Since the cost of the raw material constitutes a major part of the production cost of biodiesel, it is very important to achieve a high conversion yield. Enzymatic biodiesel would be more competitive with the chemical alternative if the reactions occurring during enzymatic alcoholysis could be fully understood and optimized.

**Fig. 1:**
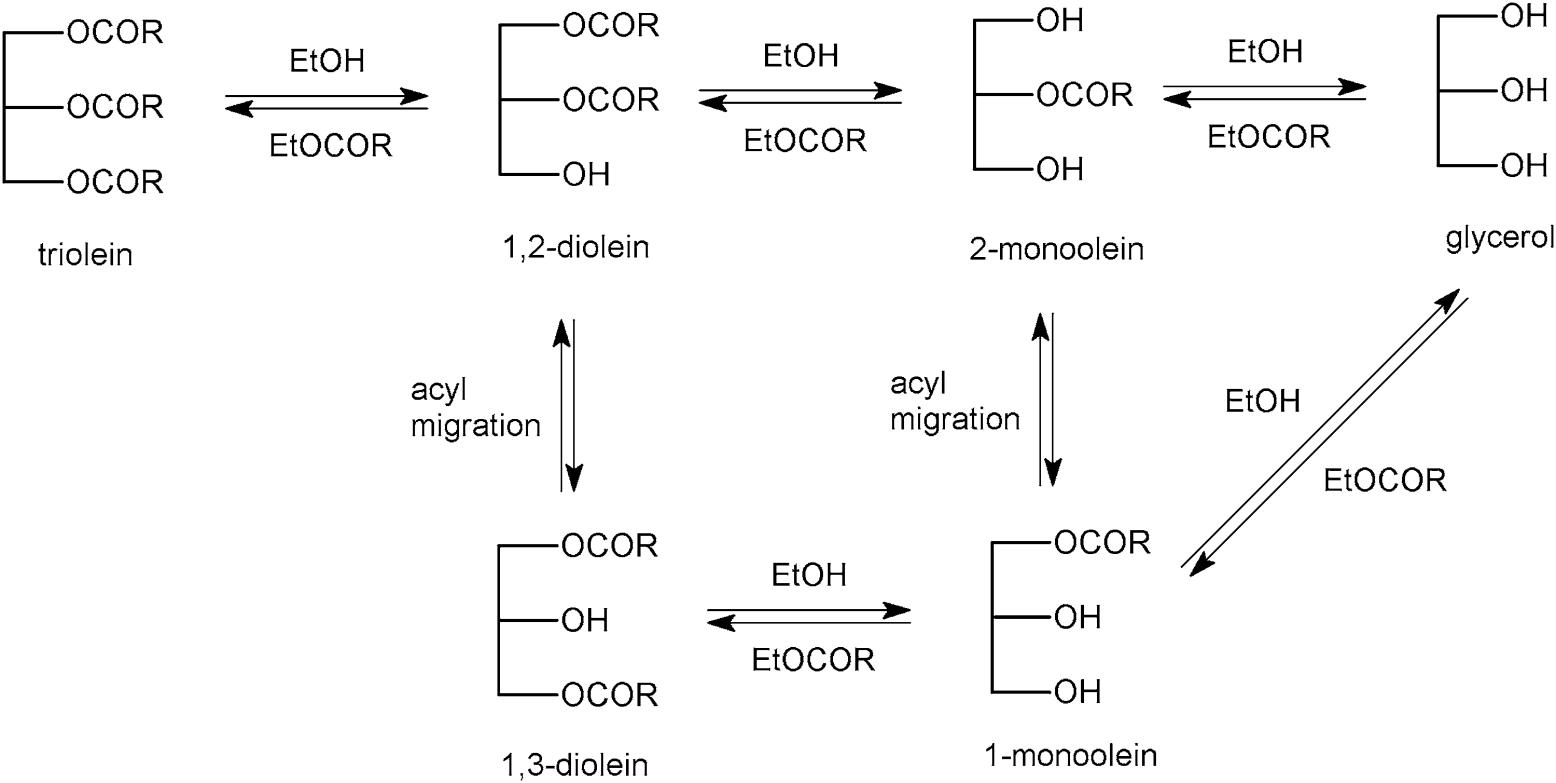
Reaction scheme for ethanolysis of triolein. All reactions except the acyl migration are lipase-catalyzed. Hydrolysis occurs to a small extent (not shown). The upper reaction sequence shows the main reactions in the conversion of triolein to ethyl oleate (EtOCOR). *RCOOH* oleic acid

In the alcoholysis of a triacylglycerol substrate, the fatty acids are removed one at a time by the lipase, resulting in alkyl esters and partial acylglycerols and, finally, glycerol, as shown in Fig. 1. The reactions occur via an acyl enzyme intermediate. Hydrolysis of this intermediate and the subsequent yielding free fatty acids is an undesired side reaction that cannot be completely avoided. Natural triacylglycerol oils contain several fatty acids that are combined in different ways in the acylglycerol molecules, resulting in a very high number of molecular species in the reaction mixture [4]. Furthermore, there are different regioisomers of the partial acylglycerols. Complete analysis of the reaction mixtures is thus very complicated and, in most cases, not practically feasible. It is, of course, important to study biodiesel production from natural fats and oils, but in order to carry out detailed studies of the reactions involved, it is preferable to study simpler model systems, ideally containing only one fatty acid.

The aim of this study was to elucidate how the regioselectivity and lipid class selectivity of lipases influence the outcome of the alcoholysis of a triacylglycerol substrate. To make detailed analyses possible, pure triolein was chosen as the substrate. Furthermore, ethanol was chosen instead of the more commonly used methanol, because it can be obtained from renewable resources, and causes less inactivation of the enzymes. Four lipases were evaluated as catalysts; they were immobilized on the same support material, rather than using the commercially available immobilized forms. This allowed a detailed comparison of the enzymes without interferences caused by different support materials. Most lipases have a preference for the 1 and 3 positions in triacylglycerol substrates, which means that the removal of the fatty acid in the 2 position could constitute a serious bottleneck in the biodiesel enzymatic process. Special attention was, therefore, paid to the analysis of the isomeric composition of the monoacylglycerols formed in the reactions, and to the effects of potential catalysts on acyl migration.

## Materials and Methods

### Chemicals

Lipase from *Candida antarctica* (Lipozyme^®^ CALB L) (CA) was kindly donated by Novozymes, and lipases from *Rhizopus arrhizus* (fine powder 9.1 U/mg)(RA), *Rhizomucor miehei* (Palatase^®^ 20000 L,≥ 20,000 U/g)(RM), and *Thermomyces lanuginosus* (Lipolase 100 L)(TL) were obtained from Sigma-Aldrich Co. (St. Louis, MO, USA). Polypropylene powder, Accurel MP1000, was purchased from Membrana GmbH (Obernburg, Germany). Methyl *t*-butyl ether (MTBE), cyclohexane, tetradecane, glycerol, and oleic acid were purchased from Merck (Darmstadt, Germany), *N*-methyl-*N*-trimethylsilylheptafluorobutyramide (MSHFBA, Machery-Nagel) from ScantecLab (Partille, Sweden), and ethanol (BDH Prolabo 95.0 and 99.5 %) from VWR (Stockholm, Sweden). Bradford reagent, *p*-nitrophenyl butyrate, and bovine serum albumin (BSA) were purchased from Sigma-Aldrich Co. (St. Louis, MO, USA), and triolein (99 %) was obtained from Larodan Fine Chemicals, Malmö, Sweden. Other chemicals were of analytical grade. All phosphate buffers were prepared using the sodium salts. The solvents were dried over molecular sieves (pore diameter 3Å, Sigma-Aldrich Co., St. Louis, MO, USA). Silica gel 60 (230–400 mesh size) was obtained from Alfa Aesar GmbH, Karlsruhe, Germany). Amberlite IR-120 (H) (BDH reagent, UK) (amberlite acidic), neutralized Amberlite IR-120 (neutralized to pH 7 by treating with 1 M NaOH) (amberlite neutral), and Milli-Q water were evaluated as acyl migration catalysts.

### Lipase Adsorption

Lipases were immobilized by adsorption onto the microporous polypropylene Accurel MP1000 (particle size 200–700 µm) using a modification of the method of Hagström et al. [5]. Different amounts of lipase powder/lipase solution were used for adsorption, depending on the protein content and the activity of the lipase preparation. The following amounts were used per gram of Accurel MP1000: CA 3.3 ml, RA 600 mg, RM 1.4 ml, and TL 1.4 ml. In all cases, the initial lipase preparation (solid or liquid) was dissolved in 10 ml sodium phosphate buffer per g support (pH 7.0, 50 mM), and added to Accurel MP1000 that was pre-wetted with ethanol (3 ml/g support). The support and the enzyme solution were incubated for 3 h on a nutating mixer at room temperature, and then filtered. The preparation was rinsed three times with 5 ml sodium phosphate buffer (pH 7.0, 50 mM), filtered, and dried overnight under reduced pressure.

### Protein Determination

Protein contents were determined by the Bradford method, using BSA as a standard [6]. The amount of lipase adsorbed onto the Accurel MP1000 was determined by measuring the amount of protein in the solution before and after adsorption.

### Lipase Activity Measurements

The hydrolytic activity of the lipase was measured using a spectrophotometric assay based on the hydrolysis of *p*-nitrophenyl butyrate (pNPB). A 20 mM stock solution of pNPB was prepared in isopropanol. The adsorption was measured continuously at a wavelength of 405 nm in a thermostat-equipped spectrophotometer at 30 °C using pH 7.0 sodium phosphate buffer. The concentration of pNPB in the cuvette was 0.4 mM, and the enzyme concentration was chosen so as to allow linear adsorbance measurements for 2 min. One unit of lipase hydrolytic activity corresponds to the amount of enzyme releasing 1 μmol of *p*-nitrophenol per minute. The hydrolytic activity immobilized on the Accurel MP1000 was estimated by measuring the lipase activity in the solution before and after adsorption.

### Preparation of Monoglycerides

#### Preparation of 2-monoolein

2-monoolein was prepared in a way similar to that described by Millqvist Fureby et al. [7]. Ethanol (600 mM) and triolein (150 mM) were dissolved in methyl *t*-butyl ether. Water (0.5 %) was added to the reaction mixture. Immobilized RA [2 % (w/v); the amount of enzyme preparation used was based on the total reaction volume] was added to the flask, which was then transferred to a reciprocal shaker and shaken for 8 h at 160 rpm and 30 °C. Samples were taken for gas chromatography (GC) analysis to monitor the reaction. The product was further purified as described by Millquist et al. [7] and crystallized from heptane at −20 °C.

#### Preparation of 1-monoolein

1-monoolein was prepared by esterifying oleic acid in excess glycerol with RA, which is a 1,3-specific lipase. Substrates were dissolved in *t*-butanol at a concentration of 0.1 M for oleic acid and 0.5 M for glycerol. Immobilized RA [2 % (w/v)] was added to the flask, which was then transferred to a reciprocal shaker and shaken for 6–7 h at 160 rpm and 30 °C. Samples were taken for GC analysis. The solvent was removed by rotary evaporation under vacuum. The oily residue was dissolved in heptane (30–35 ml heptane/g oleic acid) and centrifuged at 3,200 rpm to remove the excess glycerol and water. The supernatant, containing 1-monoolein, was incubated at −20 °C for 8 h, and the crystals were separated by vacuum filtration. The purified 1-monoolein (>98 % according to GC) was stored at −20 °C.

### Alcoholysis

The standard reaction conditions were 150 mM triolein and 600 mM ethanol in 2 ml methyl *t*-butyl ether, in a 4.5 ml glass vial capped with a septum to avoid evaporation. In monoolein alcoholysis, the concentration of monoolein was 50 mM with 200 mM ethanol. The reaction was started by adding 2 % (w/v total reaction medium) immobilized lipase preparation. The influence of potential acyl migration catalysts was studied by adding 2 % w/v of these to the reaction vial. The reactions were carried out in a thermomixer at 600 rpm at 30 °C (low enough to avoid significant enzyme inactivation) for 48 h, unless otherwise stated. Samples were withdrawn by inserting a Hamilton syringe through the septum.

The initial reaction rates were calculated as the rate of consumption of the substrate per unit weight of protein immobilized on the support (in µmol min^−1^ mg^−1^) during the first hour of the reaction. The concentration of each component was calculated as a percentage of all oleic acid equivalents initially available, taking into account the fact that diacylglycerols and triacylglycerols contain two and three fatty acid moieties, respectively. The yields of ethyl oleate are the maximum yields obtained during the reaction time.

### GC Analysis

The samples containing ethyl oleate, oleic acid, 1-monoolein, 2-monoolein, 1,2-diolein, 1,3-diolein, and triolein were analyzed by GC using a Varian gas chromatograph (430-GC-FID, Agilent Technologies Inc., Santa Clara, CA, USA) equipped with a flame ionization detector and a FactorFour™ capillary column (VF = 1 ms, length 15 m and ID 250 µm, Varian, Agilent Technologies Inc., Santa Clara, CA, USA). Helium was used as a carrier gas, and the temperature of both the injector and detector was 350 °C. The starting temperature of the column was 180 °C. This was maintained for 2.5 min and then increased to 340 °C at 10 °C/min, and then maintained for 26 min.

Samples were withdrawn from the reaction mixture and derivatized by silylation by adding an equal volume of MSHFBA and incubating them at room temperature for at least 30 min. For the analysis of solvent-free samples and solutions in *t*-butanol, the samples were diluted 10 or 5 times, respectively, before silylation. The silylation reaction was terminated by adding 99.5 % ethanol at a volume equal to that of MSHFBA. The samples were diluted 15 times with an internal standard solution in cyclohexane to obtain a sample suitable for GC in which the final concentration of tetradecane was 8 mM. The concentrations of the compounds were calculated using response factors, obtained either from theoretical molar responses [8, 9] or using a standard curve (in the case of triolein).

## Results and Discussion

The results of lipase immobilization are given in Table 1. In the case of CA and TL, most of the protein and the lipase were adsorbed onto the support. The same protein loading was seen for RA, about 38 mg/g, which means that only about one-third of the protein was adsorbed. The immobilization of lipases on porous polypropylene has been studied previously, and it has been found that Langmuir adsorption isotherms can be used to describe the adsorption process [10]. The amount of lipase on the support leading to saturation depends on the specific surface area available in the pores, and it is probable that 38 mg/g is close to the amount of lipase saturating this support [11, 12]. The fact that the amount of immobilized activity was as high as 64 % shows that RA was preferentially adsorbed compared to other proteins in the commercial lipase preparation, as has been observed previously for several lipases [10]. This means that immobilization also constitutes partial purification of the lipase. In the case of RM, almost all the was adsorbed, but only 5 % of the protein, indicating that this lipase contained a large amount of other proteins.

**Table 1:** Results of lipase immobilization on Accurel MP1000

Lipase	Immobilized activity (U/g support)	Immobilized protein (mg/g support)	Activity U/mg protein	% of activity immobilized	% of protein immobilized
*C. antarctica* B	128.7	36.2	3.6	94.3	91.3
*R. arrhizus*	155.4	37.8	4.1	63.6	35.4
*R. miehei*	23.3	0.6	38.8	90.3	5.1
*T. lanuginosus*	982.8	38.5	25.5	91.9	80.3

All activities were measured using pNPB as substrate

**Fig. 2:**
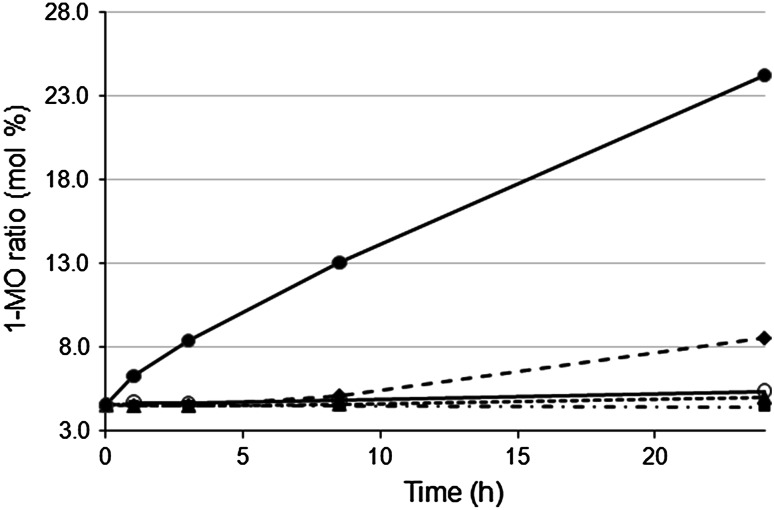
Acyl migration of 2-monoolein in MTBE with and without additives. The percentage of 1-monoolein in the total monoolein fraction is shown as a function of time. Additives: *filled circle* silica, *filled diamond* amberlite acidic, *filled triangle* water, *filled square* amberlite neutral, *unfilled circle* no additives. No lipase was used

Most lipases express some selectivity for the 1 and 3 positions in triacylglycerols, although the degree of selectivity varies. In the alcoholysis of triacylglycerols, this means that there might be an accumulation of 2-monoacylglycerols, which are only slowly converted. The addition of acyl migration catalysts to catalyze the conversion of 2-monoacylglycerols to the corresponding 1-isomers has been shown to be beneficial in improving conversion in alcoholysis [13, 14]. The effects of a number of potential catalysts on acyl migration were investigated in this study. The fastest acyl migration was observed in the presence of silica gel, as can be seen in Fig. 2. The acidic ion-exchange resin also catalyzed acyl migration, although at a lower rate, but had no effect when it was neutralized prior to use. In a previous study of isomerization of 2-monoolein, silica gel was found to be the most effective acyl migration catalyst among the solid materials tested [7], and it has also been shown to cause acyl migration in diacylglycerols [13]. Silica gel was therefore selected for further use in this study.

**Fig. 3:**
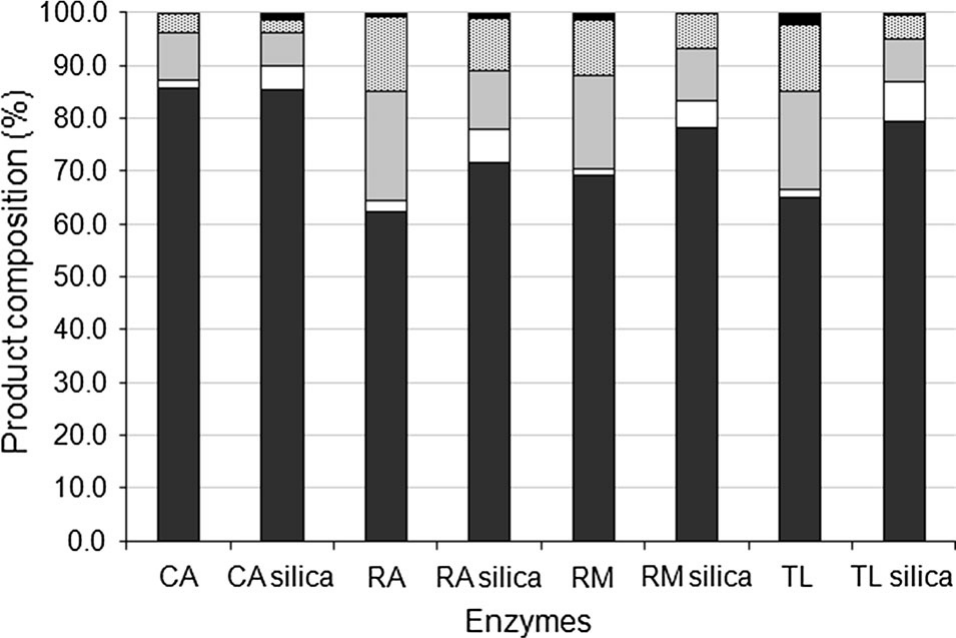
Product composition after 48 h of ethanolysis of triolein catalyzed by lipases with or without silica. *black bar* triolein, *dotted bar* diolein, *light grey bar* monoolein, *white bar* oleic acid, *dark grey bar* ethyl oleate

Alcoholysis of triolein was used as a model for biodiesel production. Silylation of the samples followed by GC analysis allowed quantification of all the reaction products. Isomers of both monoolein and diolein were separated. A typical chromatogram is shown in Fig. S1 in the supplementary material. The alcoholysis reaction was carried out with the four immobilized lipases with and without silica gel as an acyl migration catalyst. The addition of silica gel led to an increase in oleic acid formation with all the enzymes studied, and an increase in ethyl oleate yield after 48 h when using RA, RM, and TL, but not with CA, as illustrated in Fig. 3. The highest ethyl oleate yield was obtained using CA. The main side products were monoolein and diolein in all cases.

**Fig. 4:**
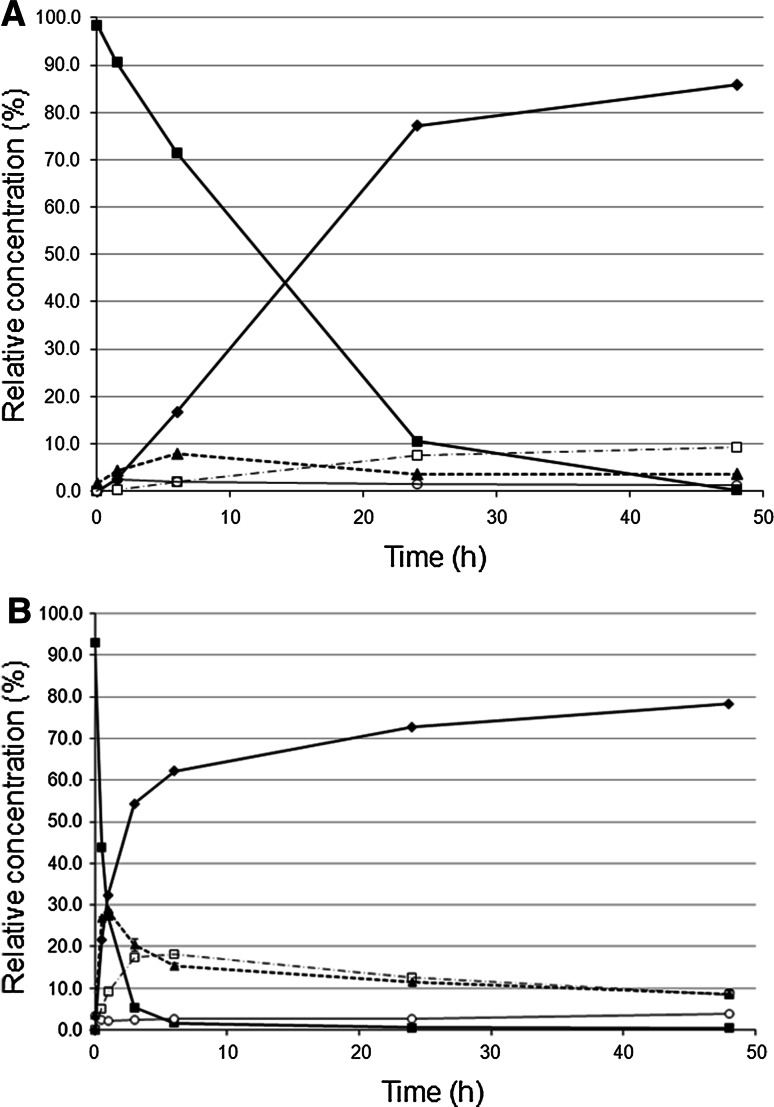
**a** Time course of triolein ethanolysis catalyzed by CA without additive. *filled diamond* ethyl oleate, *unfilled square* monoolein, *filled triangle* diolein, *unfilled circle* oleic acid, *filled square* triolein. **b** Time course of triolein ethanolysis catalyzed by TL with silica as additive. *filled diamond* ethyl oleate, *unfilled square* monoolein, filled triangle diolein, *unfilled circle* oleic acid, *filled square* triolein

Following the alcoholysis reactions showed that the triolein consumption was rather slow with CA, and that the amounts of monoolein and diolein were low throughout the duration of the reactions, as can be seen in Fig. 4a, while the ethyl oleate yield obtained after 48 h was quite high. These results indicate that CA converts triolein slowly, but converts monoolein and diolein more rapidly. Previous studies have shown that CA expresses high 1,3-specificity at high ethanol concentrations [15, 16], which would lead to accumulation of 2-monoglycerides. Apparently the moderate ethanol concentration used in the present study is favorable for complete conversion of the triacylglycerol substrate to ethyl esters, which is in agreement with previous observations [16]. Among the other lipases, the highest ethyl oleate yield was obtained with TL when silica gel was added. Rapid consumption of triolein was seen, together with the accumulation of considerable amounts of monoolein and diolein, especially during the early stages of conversion, as shown in Fig. 4b. Due to the slow conversion of monoolein and diolein, the final ethyl oleate yield was rather low.

**Fig. 5:**
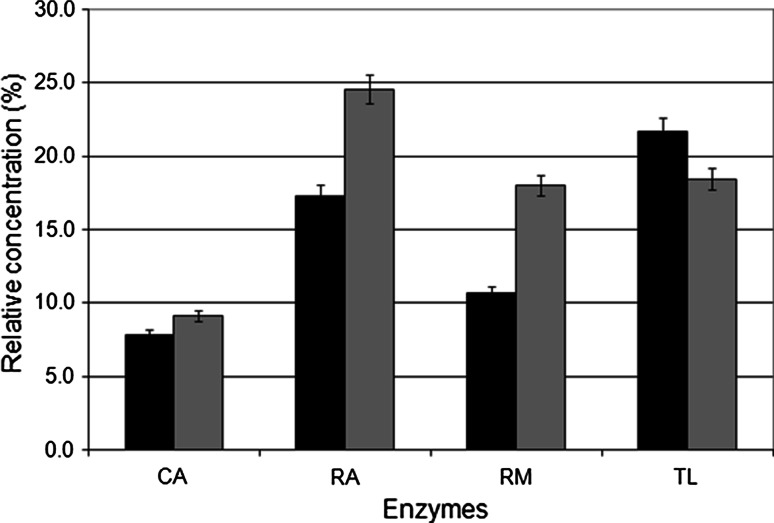
Maximal amounts of monoolein and diolein in triolein ethanolysis. The values shown are expressed as percentages of the oleic acid present in the selected substance among the total amount of oleic acid in all forms. *black bar* diolein, *grey bar* monoolein

The concentrations of monoolein and diolein showed maxima in all cases, as can be expected in consecutive reactions. The maximal amounts of monoolein and diolein provide interesting information on the relative rates of conversion of the various acylglycerol substrates by the different lipases. These values were higher for monoolein than for diolein when using CA, RA and RM, as can be seen in Fig. 5, which indicates that the third alcoholysis reaction is the slowest in these cases. Only TL provided a higher maximal concentration of diolein than monoolein. The lowest maximal values for both partial acylglycerols were obtained with CA, which is clearly very efficient in the alcoholysis of these substrates.

**Fig. 6:**
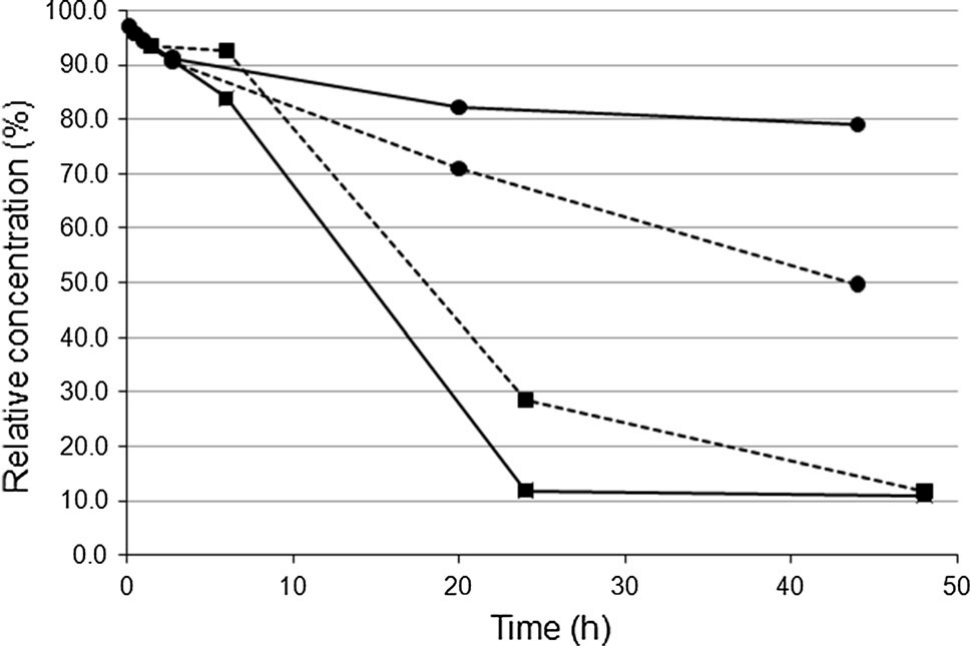
Ratio of 2-monoolein to total monoolein during triolein ethanolysis, catalyzed by CA or TL, with or without silica. *solid lines with squares* CA without additives, *dashed lines with squares* CA with silica, *solid lines with circles* TL without additives, *dashed lines with circles* TL with silica

Analysis of the regioisomer composition of the partial acylglycerols formed in the alcoholysis of triolein revealed that 2-monoolein was the dominating monoolein isomer in the initial phase in all cases. This shows that all the lipases studied have a preference for the 1 and 3 positions in triolein. During the course of the reaction, gradual conversion to the more stable 1-isomer occurred. In most cases, acyl migration was the main mechanism for this isomerization reaction. In the reaction catalyzed by TL, the addition of silica gel significantly increased the rate of acyl migration, causing a more rapid decrease in the ratio of 2-monoolein to total monoolein (see Fig. 6). However, when using CA, the addition of silica gel had the opposite effect, slowing down the decrease in the 2-monoolein:total monoolein ratio. In the CA-catalyzed reaction without silica gel, a ratio of 2-monoolein:total monoolein of about 11 % was reached after 24 h. This corresponds to the equilibrium mixture between the two regioisomers [17]. One possible explanation of these observations is that equilibration between the isomers occurred via rapid enzyme-catalyzed conversion of monoolein to glycerol and re-esterification of glycerol to monoolein, according to the scheme shown in Fig. 1. The re-esterification reaction is expected to give mainly 1-monoolein. CA might be more efficient than the other lipases in catalyzing glycerol esterification. Silica is known to absorb polar substances, including glycerol, and could thus have partly prevented the lipase-catalyzed conversion of glycerol, thereby slowing down the conversion of 2-monoolein to 1-monoolein. Similar effects were observed in the diolein fraction: 1,2 (2,3)-diolein was the dominating diolein-isomers formed by all enzymes, and CA afforded the most rapid isomerization to the more stable 1,3-isomer, a process that was partly inhibited by silica gel (results not shown). It is thus possible that the CA-catalyzed alcoholysis and re-esterification pathway also caused isomerization in the diolein fraction.

In order to obtain more direct information on the conversion of monoacylglycerols, both 2-monoolein and 1-monoolein were synthesized, and alcoholysis of these substrates was studied. Since most lipases have a preference for the 1 and 3 positions in triacylglycerols, the normal triacylglycerol alcoholysis pathway provides 2-monoacylglycerol as a key intermediate, which may accumulate. The relation between the conversion rates of 2-monoacylglycerol and triolein thus provides information on whether 2-monoacylglycerol conversion constitutes a bottleneck in the overall conversion to ethyl esters. CA converted 2-monoolein rapidly, while the opposite was observed for the other three lipases studied, as can be seen in Table 2. RA was especially slow in the conversion of 2-monoolein. These results agree with the observation that the maximal accumulated 2-monoolein concentration was much lower when using CA than the other three lipases (Fig. 5). Furthermore, a comparison with the catalytic activity observed in the hydrolysis of pNPB (Table 1) shows that there are large differences in the specificity of lipases for different substrates.

**Table 2:** Initial reaction rates using different substrates

Lipase	Initial rates of alcoholysis (µmol mg^−1^ min^−1^)			Rate ratio
	Triolein	1-monoolein	2-monoolein	2-monoolein/1-monoolein
*C. antarctica* B	0.29	1.00	1.05	1.05
*R. arrhizus*	3.75	0.89	0.11	0.12
*R. miehei*	45.69	57.50	17.64	0.31
*T. lanuginosus*	1.49	0.58	0.47	0.81

The substrate concentrations were 150 mM (triolein) or 50 mM (monoolein), lipase amount 2 % (w/v), reaction time 1 h at 30 °C

The conversion of 1-monoolein was faster than that of 2-monoolein using three of the four lipases, but there was a large variation between the enzymes (Table 2). Only RA converted 1-monoolein much faster (about 8 times) than 2-monoolein. RM converted 1-monoolein about three times faster than 2-monoolein, while the conversion rates were similar for the two isomers when using CA and TL. RA, RM, and TL are considered to be 1,3-specific lipases, which agrees with the observations that 1,2 (2,3)-diolein was the dominating (>95 %) diolein isomer (results not shown), and 2-monoolein the dominating monoolein isomer (results for TL are shown in Fig. 6) in the initial phase of our alcoholysis reactions. The results for RM and TL in Table 2 indicate that their regioselectivity is less pronounced for monoacylglycerols than for triacylglycerols, which are the normal substrates on which the classification of 1,3-specificity is based. This also shows that the efficiency of adding acyl migration catalysts to enhance biodiesel synthesis is highly dependent on which lipase is used, and significant improvements can only be expected in some cases.

Bearing in mind the considerable differences between the specificity of the lipases for different acylglycerols, it has been suggested that combinations of lipases may be beneficial in the biodiesel process. The combination of Lipozyme TL IM and Novozym 435 has been found to be beneficial in the production of biodiesel from rapeseed oil when *t*-butanol was used as a reaction medium [18]. The simultaneous use of about equal amounts of these two enzymes has also been found to be optimal for biodiesel production from lard [19]. A two-step approach, using one lipase in each step, has been described [20], where TL was used to catalyze the normal alcoholysis reaction, followed by a second step in which CA, in the form of Novozym 435, was used to catalyze the esterification of free fatty acids in the reaction mixture from the first step. The results obtained in the present study indicate that CA is the best of the enzymes studied for the conversion of diolein and monoolein, and that the other lipases are more efficient in the conversion of triolein. Therefore, a combination of RA, RM, or TL during the first phase of conversion, and CA during the second phase was evaluated. A reaction time of 24 h was chosen for the first step to achieve close to complete conversion of triolein. The second step was carried out for 48 h to get high overall conversion to ethyl oleate. The highest ethyl oleate yield was obtained with the combination of RA and CA (Table 3). RA is the most 1,3-specific lipase and is very efficient in converting triolein. It is very important that the first enzyme reduces the triolein concentration to very low levels, as CA will not decrease it much further. CA was very efficient in converting diolein, which was reduced to very low levels, while the main side product remaining was monoolein. The addition of silica gel increased the biodiesel yield slightly when TL and CA were used, but RA with CA was still the best combination. Increasing the reaction temperature from 30 to 40 °C when using the combination of RA and CA led to a further increase in yield from 94.9 to 95.9 % (Table 3). For practical applications, solvent-free systems are attractive [2, 21]. In such cases, relatively high reaction temperatures are beneficial both to decrease the viscosity of the reaction medium and to increase reaction rates. Another way to increase the reaction rate is, of course, to increase the enzyme dosage, but that adds to the process costs.

**Table 3:** Ethyl oleate synthesis using two enzymes consecutively

Relative concentrations of compounds after step 1 (%)^a^									
Enzymes used in step 1	Ethyl oleate	Oleic acid	2-monoolein	1-monoolein	1,2-diolein	1,3-diolein	Triolein	Total monoolein	Total diolein
RA	63.5	0.9	19.7	2.3	12.6	0.3	0.8	22.0	12.9
RM	61.5	0.9	15.0	2.8	11.1	0.5	8.3	17.8	11.6
TL	52.4	0.9	15.2	2.9	20.4	0.8	7.4	18.1	21.3
TL + SI	62.9	3.2	10.0	3.8	16.6	1.5	1.9	13.8	18.1
RA 40 °C	61.2	0.9	19.9	2.2	14.0	0.4	1.5	22.0	14.4

Relative concentrations of compounds after step 2 (%)^b^									
Enzymes used in step 1 and step 2	Ethyl oleate	Oleic acid	2-monoolein	1-monoolein	1,2-diolein	1,3-di olein	Triolein	Total monoolein	Total diolein
RA + CA	94.9	1.2	0.3	3.0	0.2	0.2	0.2	3.3	0.4
RM + CA	91.6	1.1	0.4	3.0	0.4	1.0	2.5	3.4	1.4
TL + CA	91.7	1.1	0.4	2.9	0.4	0.7	2.9	3.3	1.1
TL + SI + CA	93.1	2.2	0.4	2.7	0.4	0.3	0.9	3.1	0.7
RA + CA 40 °C	95.9	1.1	0.3	2.6	0.1	0.1	0.0	2.9	0.1

^a^ Step 1: 24 h with RA, RM or TL (or TL with the addition of silica gel). The reaction temperature was 30 °C except in the final case, where it was 40 °C

^b^ Step 2: 48 h with CA and two additional portions of ethanol. The reaction temperature was 30 °C, except in the final case, where it was 40 °C

## Conclusions

The lipases studied here differed considerably in their specificity with respect to both different classes of acylglycerols and to different regioisomers of acylglycerols, and this had profound effects on biodiesel formation. Interestingly, the regioselectivity of the enzymes was less pronounced for monoolein than for triolein.

The effect of silica addition on biodiesel formation was quite different depending on which lipase was used. In the reaction carried out using TL, silica gel clearly acted as an acyl migration catalyst and increased the biodiesel yield. However, the yield was reduced when silica gel was added to the reaction using CA, possibly because this additive absorbed glycerol, thereby interfering with isomerization via hydrolysis/re-esterification. Due to the differences in specificity of the lipases, it was found to be beneficial to use combinations of them, RA with CA being the most promising combination.

## Electronic supplementary material

Below is the link to the electronic supplementary material.

Supplementary material 1 (DOCX 57 kb)

## Abbreviations

BSA: Bovine serum albumin
CA: *Candida antarctica* lipase B
MSHFBA: N-methyl-N-trimethylsilylheptafluorobutyramide
MTBE: Methyl t-butyl ether
pNPB: p-nitrophenyl butyrate
RM: *Rhizomucor miehei* lipase
RA: *Rhizopus arrhizus* lipase
TL: *Thermomyces lanuginosus* lipase

## References

[1] Hernandez-Martin E, Otero C (2008). Different enzyme requirements for the synthesis of biodiesel: Novozym (R) 435 and Lipozyme (R) TL IM. Bioresour Technol.

[2] Fjerbaek L, Christensen KV, Norddahl B (2009). A review of the current state of biodiesel production using enzymatic transesterification. Biotechnol Bioeng.

[3] Adlercreutz P (2013). Immobilisation and application of lipases in organic media. Chem Soc Rev.

[4] Svensson J, Adlercreutz P (2008). Identification of triacylglycerols in the enzymatic transesterification of rapeseed and butter oil. Eur J Lipid Sci Technol.

[5] Hagstrom AEV, Nordblad M, Adlercretz P (2009). Biocatalytic polyester acrylation-process optimization and enzyme stability. Biotechnol Bioeng.

[6] Bradford MM (1976). Rapid and sensitive method for quantification of microgram quantities of protein utilizing principle of protein-dye binding. Anal Biochem.

[7] Millqvist Fureby A, Virto C, Adlercreutz P, Mattiasson B (1996). Acyl group migrations in 2-monoolein. Biocatal Biotransform.

[8] Verhaar LAT, Dewilt HGJ (1969). Gas chromatographic determination of polyhydroxy monocarbonic acids obtained by oxygenation of hexoses in aqueous alkaline solutions. J Chromatogr.

[9] Scanion JT, Willis DE (1985). Calculation of flame ionization detector relative response factors using the effective carbon number concept. J Chromatogr Sci.

[10] Gitlesen T, Bauer M, Adlercreutz P (1997). Adsorption of lipase on polypropylene powder. Biochim Biophys Acta.

[11] Barros RJ, Wehtje E, Garcia FAP, Adlercreutz P (1998). Physical characterization of porous materials and correlation with the activity of immobilized enzyme in organic medium. Biocatal Biotransform.

[12] Salis A, Sanjust E, Solinas V, Monduzzi M (2003). Characterisation of Accurel MP1004 polypropylene powder and its use as a support for lipase immobilisation. J Mol Catal B-Enzym.

[13] Du W, Xu YY, Liu DH, Li ZB (2005). Study on acyl migration in immobilized lipozyme TL-catalyzed transesterification of soybean oil for biodiesel production. J Mol Catal B-Enzym.

[14] Wang Y, Wu H, Zong MH (2008). Improvement of biodiesel production by lipozyme TL IM-catalyzed methanolysis using response surface methodology and acyl migration enhancer. Bioresour Technol.

[15] Irimescu R, Furihata K, Hata K, Iwasaki Y, Yamane T (2001). Utilization of reaction medium-dependent regiospecificity of Candida antarctica lipase (Novozym 435) for the synthesis of 1,3-dicapryloyl-2-docosahexaenoyl (or eicosapentaenoyl) glycerol. J Am Oil Chem Soc.

[16] Watanabe Y, Nagao T, Shimada Y (2009). Control of the regiospecificity of Candida antarctica lipase by polarity. New Biotechnol.

[17] Serdarevich B (1967). Glyceride isomerizations in lipid chemistry. J Am Oil Chem Soc.

[18] Li LL, Du W, Liu DH, Wang L, Li ZB (2006). Lipase-catalyzed transesterification of rapeseed oils for biodiesel production with a novel organic solvent as the reaction medium. J Mol Catal B-Enzym.

[19] Huang Y, Zheng H, Yan YJ (2010). Optimization of lipase-catalyzed transesterification of lard for biodiesel production using response surface methodology. Appl Biochem Biotech.

[20] Xu Y, Nordblad M, Woodley JM (2012). A two-stage enzymatic ethanol-based biodiesel production in a packed bed reactor. J Biotechnol.

[21] Petersson AEV, Gustafsson LM, Nordblad M, Borjesson P, Mattiasson B, Adlercreutz P (2005). Wax esters produced by solvent-free energy-efficient enzymatic synthesis and their applicability as wood coatings. Green Chem.

