# Supplementary figures and images for: Effects of Regioselectivity and Lipid Class Specificity of Lipases on Transesterification, Exemplified by Biodiesel Production
Source: J Am Oil Chem Soc. 2014 Apr 26;91(7):1283–90. doi: 10.1007/s11746-014-2465-7 (PMC4070466; doi:10.1007/s11746-014-2465-7)

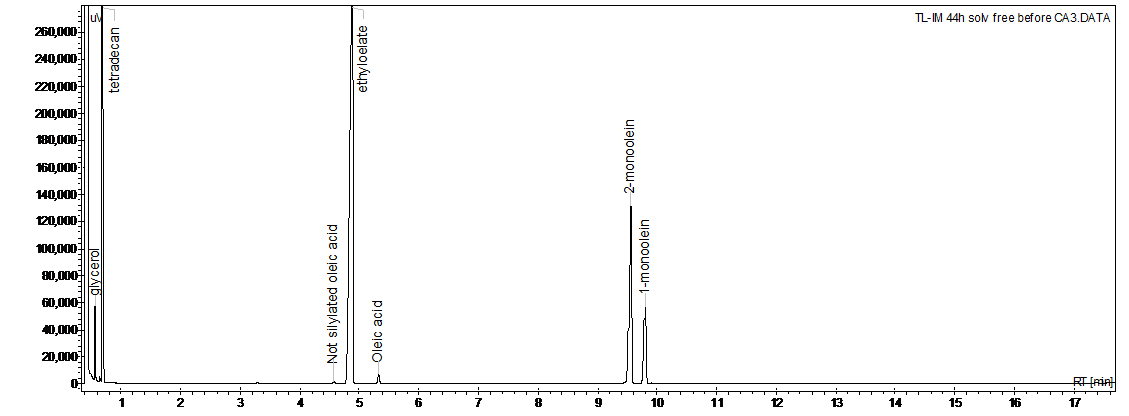


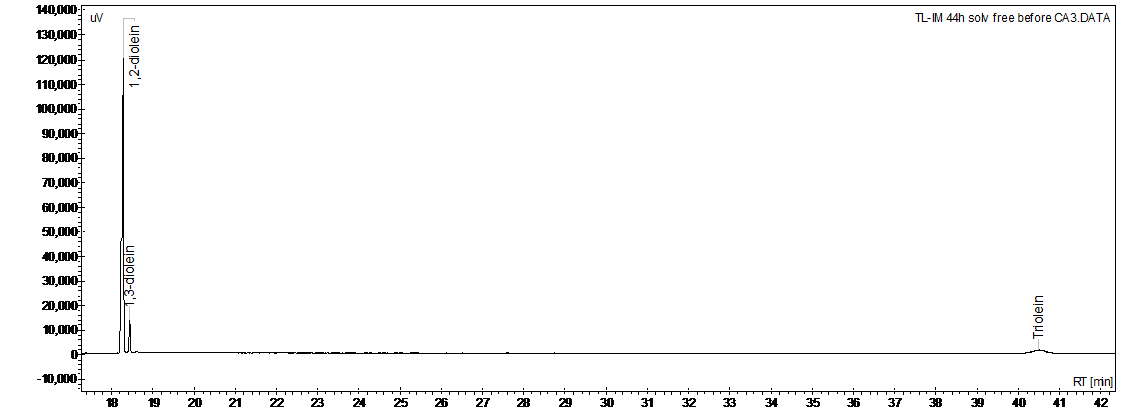


Figure S1. Typical GC chromatogram of oleic acid glycerol esters.

Supplement: Supplementary file 1 — Supplementary material 1 (DOCX 57 kb) [file 11746_2014_2465_MOESM1_ESM.docx]
